# Health seeking behavior as a predictor of healthcare utilization in a population of patients with spinal pain

**DOI:** 10.1371/journal.pone.0201348

**Published:** 2018-08-01

**Authors:** Derek Clewley, Dan Rhon, Timothy Flynn, Shane Koppenhaver, Chad Cook

**Affiliations:** 1 Rocky Mountain University of Health Professions, Provo, Utah, United States of America; 2 Duke University School of Medicine, Department of Orthopaedics, Division of Physical Therapy, Durham, North Carolina, United States of America; 3 Center for the Intrepid, San Antonio, Texas, United States of America; 4 Baylor University, Doctor of Physical Therapy Program, Waco, Texas, United States of America; 5 South College, School of Physical Therapy, Knoxville, Tennessee, United States of America; University of Nebraska Medical Center, UNITED STATES

## Abstract

**Background:**

The global burden of low back pain is growing rapidly, accompanied by increasing rates of associated healthcare utilization. Health seeking behavior (HSB) has been suggested as a mediator of healthcare utilization. The aims of this study were to: 1) develop a proxy HSB measure based on healthcare consumption patterns prior to initial consultation for spinal pain, and 2) examine associations between the proxy HSB measure and future healthcare utilization in a population of patients with spine disorders.

**Methods:**

A cohort of 1,691 patients seeking care for spinal pain at a single military hospital were included. Cluster analyses were performed for the identification of a proxy HSB measure. Logistic regression was used to identify the predictive capacity of HSB on eight different general and spine-related high healthcare utilization (upper 25%) outcomes variables.

**Results:**

The strongest proxy measure of HSB was prior primary care provider visits. In unadjusted models, HSB predicted healthcare utilization across all eight general and spine-related outcome variables. After adjusting for covariates, HSB still predicted general and spine-related healthcare utilization for most variables including total medical visits (OR = 2.48, 95%CI 1.09,3.11), total medical costs (OR = 2.72, 95%CI 2.16,3.41), and low back pain-specific costs (OR = 1.31, 95%CI 1.00,1.70).

**Conclusion:**

Health seeking behavior prior to initial consultation for spine pain was related to healthcare utilization after consultation for spine pain. HSB may be an important variable to consider when developing an individualized care plan and considering the prognosis of a patient.

## Introduction

In 2015, nearly $3 trillion USD were spent on healthcare in the United States. Healthcare spending has consistently outpaced growth in annual income[1], representing 17.8% of the gross domestic product.[2] To some extent, increased spending is explained by accumulative chronic disease associated with lifestyle, and an increasingly aging population.[2] However, healthcare is not consumed uniformly across the population. Specifically, five percent (5%) of the U.S. population consumes 50% of all healthcare spending, and 25% of the population is responsible for 86% of the total.[3]

A large portion of healthcare utilization comes from individuals with low back and neck pain.[4] The number of annual visits to a physician in the United States for low back or neck pain exceeds $52 million USD and the estimated direct medical costs associated with these conditions surpasses $250 billion USD annually.[4] These values likely underestimate the true costs of these conditions because they do not factor in the indirect costs, such as lost work productivity, and secondary downstream healthcare effects. Individualized health seeking patterns or behaviors, conceptualized as ‘how the patient interfaces with the healthcare system and how often’are known to drive visits, costs, and type of care provided.[5]

A behavioral model of healthcare utilization was developed in the 1970s by Andersen, and continues to evolve.[6–8] In this model, healthcare utilization is described as the quantity of healthcare services used; a concept that can be measured by costs and visits.[9] In contrast, health seeking behavior (HSB) is the *behavioral* component that drives healthcare utilization. Conceptually, HSBs are mediated by predisposing factors (e.g., age, sex, cultural, ethnic, and social factors), enabling factors (e.g., financial, organizational, and access to care), and need factors (e.g., both the patient and the medical provider’s view and experiences).[7] Each of these factors is postulated to influence an individual’s decision to seek initial and continued care for their perceived health status. It may seem logical that the utilization of healthcare resources is a direct measure of the severity of the condition. In other words, the more serious or involved the medical condition is, the greater the amount of healthcare services used. For instance, metastatic cancer may result in a long drawn out pathway of care with many different medical services while a mild ankle sprain might be satisfied with a single visit to a primary care provider. However, this is often not the reality as other factors beyond the severity or complexity of the disease are often stronger predictors of healthcare utilization.[10]

There is a dearth of studies related to musculoskeletal conditions that have investigated HSB and health related outcomes, including healthcare utilization. The paucity of research on HSB, especially for musculoskeletal disorders, is likely related to the fact that a consensus for measuring HSB does not exist. Some studies have used attitudinal or behavioral-based questionnaires to ascertain the level of HSB for chronic pain populations[11, 12]; however, the applicability of these tools has not been validated in populations with spine pain. Others[12] have used a proxy measure that is based on cumulative healthcare consumption (i.e., costs and visits) *and* types of healthcare consumption (i.e., provider and setting types). These proxy measures tend to lack sophistication, and do not measure the behavioral aspect of healthcare seeking. A proxy measure of HSB that encapsulates the behavioral components is essential to facilitate further study of this construct. The exploration of a HSB construct is timely and important and allows an understanding of factors beyond disease severity that drive healthcare spending. Healthcare providers would also benefit from an understanding of which individuals and conditions might be associated with higher volumes of healthcare utilization.[13] Therefore, the aims of this study were to: 1) develop a proxy measure of HSB based on healthcare utilization patterns and 2) examine associations between the developed proxy HSB measure and future healthcare utilization in a population of patients with low back and neck disorders. We explored these objectives by using a contained, single payer, health system data repository that included a large number of healthcare utilization variables. We hypothesized that those who were identified as being high health seekers before the first medical visit for a spinal disorder would also be those responsible for the majority of healthcare utilization (i.e., top 25%) after the initial visit.

## Methods

### Selection of sample

The Military Management Analysis and Reporting Tool (M2) was used to source the data, and services approximately ten million beneficiaries around the world. Data are collected, validated and processed into this centralized and proprietary database, with regulatory oversight by the US Defense Health Agency (DHA). All beneficiaries captured within M2 have a unique person identifier that is common to all data files. This allows for merging of single person level variables longitudinally, across many data sources. Veracity of data is accomplished through a validated internal process using robust imputation algorithms to address data errors.[14]

**Fig 1:**
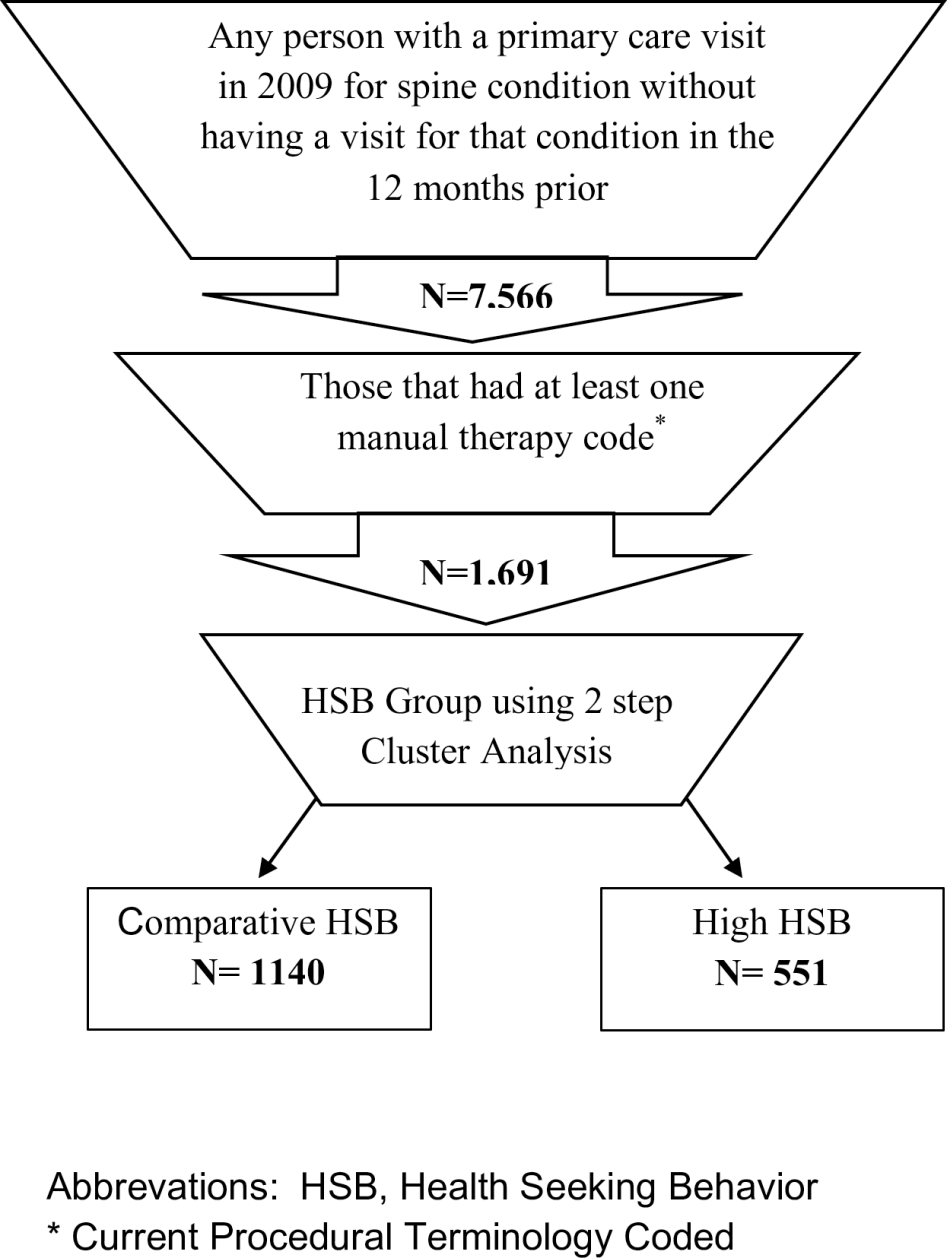
Study flow.

We utilized a cohort of patients seeking care in a single Military Hospital in Tacoma, WA, for their initial consultation for a spine disorder (i.e., low back or neck pain) between January 1 and December 31, 2009. Unlike many other health systems in the U.S., patients in this setting have no copay or insurance limitations. Patients were eligible for inclusion if they had not sought care for a spine condition during the preceding 12 months. To improve the homogeneity of the sample, we chose a cohort with spinal pain that had also received at least one manual therapy treatment (Current Procedural Terminology codes 97140, 98925 to 98929, and 98940 to 98943) as part of their care. Because treatment selection is generally dictated by clinical presentation,[15, 16] this allowed us to better isolate a group with more similar symptoms and who received similar treatment strategies as part of their plan of care. Data reflecting healthcare utilization were collected for the 12 months before and after the *indexed visit* (initial visit for low back or neck pain). The M2 includes all data from outpatient and inpatient settings, prescription medications, and imaging procedures. The process for selection of the cohort is demonstrated in Fig 1. Age, sex, military beneficiary category (active duty, retired, or dependent), condition, and comorbidities were also collected. Economic variables (e.g., costs and visits) across a large number of settings and provider types were also collected.

All patient information was fully anonymized in the copy of the dataset used for anaysis. Ethics approval was provided by the Army Western Regional Medical Command Institutional Review Board. All study procedures and analyses were compliant with the Health Insurance Portability and Accountability Act.

### Aim One: Development of a proxy measure of health seeking behavior (pre-index data)

#### Proxy Variable Conceptualization

Our goal was to create a proxy measure that was based on healthcare consumption prior to the index date. To reflect these constructs, we qualitatively evaluated a number of potential variables within M2 that reflected both concepts. Because we attempted to capture the behavioral aspect of healthcare utilization, we targeted variables that were patient-influenced, representing actions taken to rectify perceived ill-health (e.g., emergency department or urgent care visits). Another consideration for the cluster inputs were the various medical disciplines (e.g., primary care). Other potential variables included cumulative healthcare consumption (total costs and visits, spine related costs, medications used, and imaging costs). Ultimately, the variables considered were total primary care visits, total primary care costs, total emergency or urgent care costs, total emergency or urgent care visits, spine related costs, spine related visits, medication use, and diagnostic imaging procedures. Specialist care referrals are often dictated by the general practitioner, and less likely to be influenced by patient behavior, so the focus was on settings which all patients could access equally (general practice visits in primary care and emergency department). We inputted each of these variables independently and in various combinations.

#### Data analysis

To determine the most robust, structured proxy measure of HSB, we used a fixed two-group two-step cluster analysis to split the dataset into distinct groups based on significant criterion variables. We evaluated multiple models that were based on the proxy variable conceptualization to identify the strongest cluster. Two-step cluster analysis is a model used when working with large datasets to identify subgroups based on selected criterion variables,[17] allowing for inclusion of both continuous and categorical variables. When considering inputs for cluster analysis, the strength is determined by silhouette, which is a measure of consistency within clusters of data.[17] Measured values range between -1 and +1, with values closer to one being optimal.[17] The more closely a case is matched to its identified cluster group, and the more poorly it is matched to its neighboring group, the stronger the cluster. Because two distinct cluster groups were identified, we compared and reported the differences between the groups.

### Aim Two: Determine association between health seeking behavior and future healthcare utilization (post-index data)

#### Outcome variables

We captured various types of healthcare utilization outcomes in order to better capture true healthcare consumption. Healthcare utilization can be measured in cost or visit units across various provider, setting, or diagnostic categories. The primary healthcare utilization outcomes of interest were total outpatient costs and visits, and we calculated these by aggregating all post index outpatient visits and their related costs. We were interested in further examining healthcare utilization, specific to those providers or settings that served as health system access points (i.e., primary care, emergency room or urgent care). We used the same approach for both post-index primary care and emergency room costs or visits and total medical costs or visits. We were also interested in healthcare utilization directly attributed to low back or neck pain. We were able to aggregate post-index direct costs associated with these conditions. In total, we created eight healthcare utilization categories (total outpatient costs and visits, primary care costs and visits, Emergency-room / urgent care costs and visits, lumbar costs, and cervical costs). In order to capture high healthcare utilization across all eight outcomes, we dichotomized each one based on the *Agency for Healthcare Research and Quality* (AHRQ) report determining that 25% of healthcare consumers were responsible for 86% of healthcare spending.[3] We calculated a dichotomous variable for each of the healthcare utilization categories by running separate frequency tables for each one, and splitting them by quartile rank so that the top 25% were considered high healthcare utilizers, and the remaining were the control group.

#### Covariates

A number of potential covariates existed in the dataset that could explain high healthcare utilization and influence the results. These were identified as age, sex, military beneficiary category (i.e., active duty, dependent, retired, or other), comorbidities, and alcohol or tobacco use. Comorbidities were identified based on diagnostic codes entered by medical providers in electronic medical records. The list of specific codes used in this cohort has been published,[18] and includes metabolic syndromes, mental health disorders, chronic pain diagnosis, and cardiovascular disease. While all comorbidities and alcohol or tobacco use in this dataset could be measured as continuous data, the variability in diagnostic coding by each healthcare provider necessitated dichotomization. Therefore, if any patient received a diagnostic code for any of the comorbidity categories, or alcohol and tobacco use, at any point prior to index, they were considered as having the comorbidity.

#### Data analysis

Logistic regression models were performed, both adjusted and unadjusted. An unadjusted binomial logistic regression model was run for 8 variables used to identify the predictive capacity of pre-index HSB on each of the eight healthcare utilization outcomes. Multicollinearity of the independent variables was assessed using variable inflation factor and tolerance assessment. We used adjusted multivariate logistic regression to account for the covariates of age, sex, military beneficiary category, and comorbidities. We calculated Nagelkerke R Square, which approximates the proportion of variance in the dependent variable associated with the predictor (independent) variables.[19] The closer the value is to 100 percent, the better the model.[19] All data were analyzed using IBM SPSS version 24.0 (IBM Corp, Armonk New York).

## Results

### Full sample characteristics

After eligibility assessment, 1,691 patients were included in the final cohort. The average number of primary care visits for all patients was 6.82 (SD = 6.25). Mean total healthcare visits were 16.13 (SD = 18.35) and mean total healthcare costs were $7,043.14 (SD = $15,490.76). The sample was primarily male (N = 961; 56.8%), and the mean age was 36.8 years (SD = 11.0). The majority of the sample was active duty (66%), followed by dependents (16%), and retired service members (8%). The most common physical comorbidities were sleep disorders (N = 354; 20.9%) and hypertension (N = 311; 18.4%). The most common mental health comorbidities were anxiety (N = 334; 19.8%) and depression (N = 313; 18.5%).

#### Aim One: The proxy measure of health seeking behavior

We evaluated a large number of two-step cluster models, and the strongest model included only one variable input: primary care visits for the 12 months preceding the onset of low back or neck pain. Using only pre-index primary care visits as the input variable, two-step cluster analysis identified two groups of health seekers. This resulted in a 0.7 silhouette score, indicating a strong cluster with good cohesion and internal consistency.[17] This calculated cluster variable served as the proxy measure of HSB. Those categorized as high HSBs made up 32.6% of the sample (n = 551). Specific to the two-step cluster input variable of primary care visits, the high HSB group had 13.61 (SD = 6.52) primary care visits while the comparator health seeking group had only 3.54 (SD = 2.11). See Table 1 for additional comparisons between the two groups of health seekers including healthcare utilization, demographics, and comorbidities.

**Table 1:** Comparison of baseline descriptive data prior to consultation for spine pain.

Variable	Comparator Health Seeking Behavior (N = 1140)	High Health Seeking Behavior (N = 551)	P value
Pre-Index Healthcare Utilization [Mean (SD) ]			
Number of Primary Care Visits	3.54 (2.11)	13.61 (6.52)	P<0.01
Number of Unique Medications	5.48 (4.91)	14.46 (8.16)	P<0.01
Number of Unique ICD-9 Diagnosis Codes	12.91 (9.27)	29.86 (15.35)	P<0.01
Number of Healthcare Visits	9.73 (10.52)	29.38 (23.33)	P<0.01
Healthcare Costs	$2,833.14 ($3,834.35)	$9,251.21 ($10,955.98)	P<0.01
Demographic Information			
Number of patients in Beneficiary Category N(%)			
Active Duty	727 (63.8%)	390 (70.8%)	P<0.01
Dependent	189 (16.6%)	81 (14.7%)	P<0.01
Retired	109 (9.6%)	24 (4.4%)	P<0.01
All others	115 (10.1%)	56 (10.2%)	P<0.01
Mean Age in Years (SD)	37.36 (10.65)	35.61 (11.67)	P<0.01
Female Gender N(%)	444 (38.9%)	286 (51.9%)	P<0.01
Number of Patients with Physical Comorbidities N (%)			
Obesity	152 (13.3%)	115 (20.9%)	P<0.01
Tobacco use	217 (19.0%)	139 (25.2%)	P<0.01
Chronic pain	86 (7.5%)	77 (14.0%)	P<0.01
Hypertension	194 (17.0%)	117 (21.2%)	P = 0.04
Rheumatoid arthritis	2 (0.2%)	2 (0.2%)	P = 0.50
Myalgia	71 (6.2%)	54 (9.8%)	P<0.01
Osteoporosis	63 (5.5%)	42 (7.6%)	p<0.01
Sleep disturbances	202 (17.7%)	152 (13.3%)	P<0.01
Diabetes	32 (2.8%)	22 (4.0%)	P<0.19
Number of Patients with Mental Health Comorbidities N(%)			
Mood disorders	49 (4.3%)	41 (7.4%)	P<0.01
Nonorganic psychosis	0 (0.0%)	3 (0.5%)	P = 0.01
Anxiety, dissociative, and somatoform disorders	178 (15.6%)	176 (31.9%)	P<0.01
Personality disorders	6 (0.5%)	21 (3.8%)	P<0.01
Nonorganic sleep disorder	92 (8.1%)	81 (14.7%)	P<0.01
Pain related to psychological factors	13 (11.4%)	9 (1.63%)	P = 0.40
Acute reaction to stress	14 (1.2%)	12 (2.2%)	P = 0.14
Adjustment reaction	204 (17.9%)	177 (32.1%)	P<0.01
Depressive disorders	162 (14.2%)	151 (27.4%)	P<0.01

Abbreviations: SD, Standard Deviation; ICD-9, International Classification of Disease 9^th^ Edition

#### Aim Two: The association between health seeking behavior and future healthcare utilization

**Unadjusted analyses.** Unadjusted binomial logistic regression models were run for 8 variables to determine the predictive capacity of our proxy measure of HSB on healthcare utilization after initial consultation for low back or neck pain. HSB predicted high healthcare utilization for all cost types, including total outpatient costs, primary care costs, emergency and urgent care costs, low back pain related costs, and neck pain costs. HSB also predicted high healthcare utilization for all visit types, including total outpatient visits, primary care visits, and emergency or urgent care visits. (Table 2)

**Table 2:** Unadjusted binomial regression of predictor variables and healthcare utilization.

Health Services Utilization	Odds Ratio (95% CI)	P Value	Nagelkerke
**Healthcare Costs**			
Total Outpatient	2.72 (2.16, 3.41)	P<0.01	6.30%
Primary Care	2.87 (2.28, 3.60)	P<0.01	7.00%
ER / Urgent Care	2.22 (1.77, 2.78)	P<0.01	4.10%
Lumbar	1.54 (1.23, 1.94)	P<0.01	1.20%
Cervical	1.35 (1.07, 1.69)	P = 0.01	0.60%
**Healthcare Visits**			
Total Outpatient	2.48 (1.98, 3.11)	P<0.01	5.30%
Primary Care	3.16 (2.53, 3.94)	P<0.01	8.50%
ER / Urgent Care	2.41 (1.86, 3.13)	P<0.01	4.30%

Abbreviations: CI, Confidence Interval; ER, Emergency Room

**Adjusted analyses.** After running the adjusted multinomial logistic regression, our proxy HSB variable was still predictive of high healthcare utilization for low back pain costs but not for neck pain costs. HSB did predict total healthcare costs, primary care costs, and emergency or urgent care costs. Additionally, HSB predicted high healthcare utilization for outpatient visits, primary care visits, and emergency or urgent care visits. (Table 3)

**Table 3:** Multinomial regression analysis of predictor variables and healthcare utilization outcomes adjusted for age, sex, military beneficiary category, comorbidities, and alcohol and tobacco use.

Health Services Utilization	Adjusted Odds Ratio (95% CI)	P Value	Nagelkerke
**Healthcare Costs**			
Total Outpatient	1.93 (1.50, 2.50)	**P<0.01**	16.90%
Primary Care	2.05 (1.60, 2.63)	**P<0.01**	12.30%
ER / Urgent care	1.68 (1.31, 2.16)	**P<0.01**	9.10%
Lumbar	1.31 (1.00, 1.70)	**P = 0.05**	12.90%
Cervical	1.18 (0.91, 1.52)	P = 0.22	6.50%
**Healthcare Visits**			
Total outpatient	1.94 (1.49, 2.52)	**P<0.01**	20.70%
Primary Care	2.28 (1.78, 2.91)	**p<0.01**	15.60%
ER / Urgent care	1.81 (1.36, 2.42)	**p<0.01**	11.00%

OR: CI: Confidence Interval, ER: Emergency Room

## Discussion

There were two aims for this study. The first was to develop a proxy measure of HSB based on cumulative *and* type of healthcare consumption patterns before initial consultation for low back or neck pain. The second was to determine if there was an association between the proxy HSB measure derived from the initial aim and future healthcare utilization, in patients with low back or neck pain. Using a two-step cluster analysis, we were able to develop a proxy measure of HSB based on cumulative primary care visits for the 12 months preceding the onset of low back or neck pain. This proxy measurement of HSB predicted all eight post-index high healthcare utilization outcomes.

### Aim one: The proxy measure of health seeking behavior

A critical part of this study was the development of a HSB measure. Potentially, the most compelling reason for the poor investigation of HSB in musculoskeletal conditions to date is that the measurement of HSB has not been not well defined. We endeavored to create a proxy measure of HSB that can be replicated within other administrative databases. For our two-step cluster analyses, we considered only variables that were more likely to be mediated by patient behaviors because they have the greatest potential for modification. After assessment of a number of potential cluster models, we identified primary care visits prior to onset of neck or low back pain as the strongest representative input variable for the measurement of HSB. We found that those who were considered to be high healthcare utilizers after the index visit had four times the amount of primary care visits in the year leading up to initial consultation for neck or low back pain.

Contextually, the use of primary care visits as a single input for cluster analysis is well supported in the literature. While there are many care providers for the management of low back and neck pain, primary care is typically the entry point into the healthcare system for most individuals.[13–15] The primary care provider is known as the “gatekeeper” in most medical environments and their referral patterns have been previously studied.[20] Further, multidisciplinary guidelines for spine pain are often geared toward primary care physician management[21] and are considered influential in downstream healthcare use.[22] For nonspecific spine pain, primary care provider guidelines advise a conservative approach to care. Being concordant with these guidelines has been associated with lower overall healthcare utilization.[13]

### Aim two: The association between health seeking behavior and future healthcare utilization

Using the identified proxy measure of HSB, we found that those who were considered high health seekers before initial consultation for spine pain had a two and a half to three times greater risk of being high healthcare utilizers after consultation for spine pain, based on outpatient visits *and* costs, primary care visits *and* costs, and emergency room visits *and* costs. Health seeking behavior also predicted direct costs related to spine pain. The fact that direct neck pain costs were not predicted by HSB after adjusting for covariates was not surprising, and this finding supports research that demonstrates that high downstream healthcare utilization has a strong association with the initial provider consulted.[23]

Enabling health seeking behavior, as described by Andersen,[7] may be inadvertently promoted in the approach clinicians take to managing patients. For example, a clinician might advise someone with a new onset of severe low back pain to pursue continual (or new provider) services until a diagnosis has been identified. This may include referral for advanced imaging procedures and high cost treatment approaches. While well-intentioned, this is an example of mismanagement, leading to a guideline discordant pursuit of a diagnosis, which may foster unnecessary care seeking, and has been shown to increase healthcare utilization with no value added.[13] One possible explanation for this type of guideline discordant care relates to patient satisfaction. Healthcare providers naturally have an innate desire to satisfy their patients. Moreover, patient satisfaction is often used as a primary outcome measure, and even tied to reimbursement incentives.[24] This approach tips the focus of care towards engaging patient satisfaction rather than educating and redirecting expectations, which align with guideline-concordant care. This faulty management system, while providing short-term satisfaction, can result in more harm downstream (including increased healthcare utilization and high-risk procedures without changes in outcome).[25] The problem is exacerbated even more if the patient has a behavioral propensity to seek healthcare at higher rates.

Equally important to identifying a proxy measure of HSB is determining a valid measure for healthcare utilization. Healthcare utilization has been classically defined as the outcome of the interaction between the healthcare provider and the patient; in other words, the costs and visits associated with each type of healthcare entity.[26] In this database, we identified eight different types of healthcare utilization categories that could serve as the outcome variable. We elected to assess both costs *and* visits to gain a comprehensive picture of healthcare utilization. More importantly was the way we defined high healthcare utilization. It has been termed healthcare super utilization;[27] however, this term has not been consistently defined across studies. Depending on the study, super utilization rates range from the top one to top ten percent (or more) of healthcare costs.[27] Complicating the issue, the AHRQ Medical Expenditure Panel Survey report has varying classifications of high utilization rates ranging from the top one to top 25 percent of healthcare consumers.[3] We elected to use the top 25 percent (which is reported to account for 86% of total healthcare costs) primarily because we were more interested in high utilization versus super utilization. Furthemore, we wanted to capture a larger breadth of healthcare utilizers and thereby reduce the influence of multiple morbidity as the primary driver of healthcare utilization.[3, 28]

Future healthcare utilization after the onset of spine pain has been researched extensively.[23, 29] A number of predictors of future healthcare utilization related to these conditions have been identified.[23, 30, 31] Some of the known predictors of future healthcare utilization include baseline health status (including physical and mental health comorbidities), age, sex, condition chronicity, access to care, care pathways (which provider is seen first), and as previously mentioned, guideline adherence.[32–40] Past healthcare costs are also a powerful predictor of future healthcare costs. There are a number of instances in the literature that have demonstrated the predictive capacity of future healthcare utilization based on prior healthcare utilization. For example, a study by Bertsimas et al.,[41] found that two years of prior healthcare costs were associated with the future year of healthcare utilization. Furthermore, in a sample of workers with a recent onset of neck pain, patterns of healthcare utilization prior to injury have also been associated with increased healthcare costs.[29] Studies that investigate pre-consultation healthcare utilization are important in developing a better understanding of how individuals historically interface with the health system and the influence on future utilization. The results from our study not only support the notion that previous healthcare utilization predicts future utilization, but that the behavioral aspects of healthcare seeking need to be considered.

The findings related to urgent care in our study were of particular interest. Emergency room super-utilizers are categorized as individuals that frequent the emergency room. Our cut-point for emergency room and urgent care was only one visit. This is dramatically different than other studies that have investigated super users.[27, 42–44] One explanation for the differences between emergency department utilization rates between our study and previous studies is that we used the top 25 percent as the cut point for high healthcare utilization. If we had used the top five or one percent AHRQ values, we would have likely obtained more information about super utilizers versus high utilizers of healthcare. As such, the number of emergency room or urgent care visits in that super utilizer group would have been greater than our results indicated. Another explanation for the differences in emergency department utilization goes back to patient satisfaction. Health systems that have high patient satisfaction ratings tend to have decreased utilization of emergency room services[45], but instead have higher utilization of outpatient services.[45] We do not know the patient satisfaction ratings in our cohort for comparison.

### Clinical implications

Human behavior is complex, and HSB, much like other types of behaviors, is affected by consequences.[46] As people react to a stimulus (i.e., pain), they are rewarded (or punished), which helps shape future behavior. The behavior of health seeking can be especially rewarded in a free market health system, where reassurance or diagnostic labels can be pursued continuously. While there appears to be a clinical awareness of HSB,[47] clinicians should obtain information from their patients regarding these behavioral attributes, including prior healthcare utilization, especially for rendered services not associated with the condition they are consulting about. For instance, if a patient is seeking healthcare services for neck or back pain, we suggest addressing the number *and* type of total medical visits the patient has sought care for during the preceding year, beyond only neck or back pain-related healthcare services. To minimize the risk of the provider enabling health seeking behaviors in patients, guideline concordant care is essential. This includes educating patients on the value of guideline concordant care, and the downside to low-value care, and by limiting the overuse of expensive diagnostic imaging and frequently invasive treatment strategies that are ineffective, or associated with negative long term outcomes.

### Study limitations

First of all, while we feel our proxy measure of HSB was appropriate, we were not able to capture all of the complexities associated with HSB. As previously mentioned, behavior is complex, and using a quantitative pattern of healthcare utilization as a measure of HSB only provides a preliminary understanding of the construct. There are likely other variables not reflected in this healthcare database that influence HSB. Furthermore, although we were able to categorically define HSB, it is likely that the construct is a continuous attribute. Furthermore, the study sample came from a military hospital, where access to care is arguably very different than in other health systems, and therefore these results may not be generalized to other settings (higher rates of military service service members and males). Because of good access to care in the MHS, the results from this study are presumably skewed toward higher overall healthcare utilization rates. In the Andersen behavioral model of healthcare utilization, access to care is one of the components that should be considered.[6–8] Nevertheless, it could be argued that because we used a sample where access to care issues were mitigated, the results may have a higher representation of behavioral components that are less influenced by external factors. Finally, we were interested in healthcare utilization as the only outcome in this study. While understanding healthcare utilization is especially important in an evolving healthcare landscape, we did not capture clinically important information such as self-reported changes in functional status, disability, or pain. Therefore, from a clinical perspective, we cannot conclude whether or not the healthcare services that were rendered were justified or led to clinically meaningful change for the patient. The evidence supports the notion that healthcare spending is more of a product of supply sensitive services, and not a reflection of proven benefit, including function.[48] Despite these limitations, this preliminary evidence is of interest and can serve to establish hypotheses for future prospective trials.

### Future research

Health seeking behavior is an emerging construct that must be considered in the management of musculoskeletal disorders, especially in light of the growing chronic pain burden. Due to its complexity, additional research is greatly needed, specifically to look at the impact of HSB on a variety of indexed events and patient-reported outcome measures. Furthermore, using a similar model, the impact of HSB on other healthcare services, including downstream utilization of non-pharmacological and pharmacological management strategies should be further investigated.

## Conclusion

Patients considered to be high health seekers prior to initial consultation for spine pain were also most likely to have the highest amount of healthcare utilization after initial consultation. The developed proxy measure of HSB based on primary care visits prior to initial consultation predicted high healthcare utilization (top 25% healthcare visits and costs). These findings provide insight into the predictive capacity of health seeking patterns for musculoskeletal conditions.
